# Metaphedrone (3-Methylmethcathinone): Pharmacological, Clinical, and Toxicological Profile

**DOI:** 10.3390/medicina60030466

**Published:** 2024-03-12

**Authors:** Igor Kelečević, Ana-Marija Vejnović, Jovan Javorac, Nemanja Gvozdenović, Nataša Janjić, Vesna Mijatović Jovin

**Affiliations:** 1 Clinic for Psychiatry, Clinical Center of Vojvodina, 21000 Novi Sad, Serbia; 2 Faculty of Medicine, University of Novi Sad, 21000 Novi Sad, Serbia; 3 Department of Psychiatry and Psychological Medicine, Faculty of Medicine, University of Novi Sad, 21000 Novi Sad, Serbia; 4 Institute for Pulmonary Diseases of Vojvodina, 21204 Sremska Kamenica, Serbia; 5 Department of Internal Medicine, Faculty of Medicine, University of Novi Sad, 21000 Novi Sad, Serbia; 6 Department of Emergeny Medicine, Faculty of Medicine, University of Novi Sad, 21000 Novi Sad, Serbia; 7 Clinic for Orthopedic Surgery and Traumatology, Clinical Center of Vojvodina, 21000 Novi Sad, Serbia; 8 Department of Surgery, Faculty of Medicine, University of Novi Sad, 21000 Novi Sad, Serbia; 9 Department of Pharmacology, Toxicology and Clinical Pharmacology, Faculty of Medicine, University of Novi Sad, 21000 Novi Sad, Serbia

**Keywords:** metaphedrone, 3-methylmethcathinone, 3-MMC, synthetic cathinones, intoxication

## Abstract

*Introduction:* Synthetic cathinones are a group of novel psychoactive substances used as an alternative to classical recreational drugs. As a result of legal prohibitions on older generations of these compounds, new formulations appeared on the drug market. One of them is metaphedrone (3-methylmethcathinone, 3-MMC), a structural isomer of 4-methylmethcathinone and a psychostimulant drug. Metaphedrone became popular in a large number of countries in a short period of time. *The aim:* The collection, analysis, and review of relevant research on the subject of metaphedrone in order to present information about the pharmacological, clinical, and toxicological profile of this compound. An assessment of the significance and role of metaphedrone in consumption patterns of novel psychoactive substances among recreational drug users. *Methodology:* By using search engines like Google Scholar and PubMed, the relevant literature on metaphedrone was looked for and analyzed. The search was not limited to a specific period of time. All information regarding the compound of interest was analyzed and presented. *Key results and discussion:* All novel psychoactive substances are abused due to their pronounced stimulatory, hallucinogenic, dissociative, and euphoric and/or relaxing characteristics. Users of 3-methylmethcathinone usually opt for this substance for recreational purposes and/or sexual stimulation. Metaphedrone has the potential to cause a psychological dependence to the users. It was determined in relevant studies that most users are from 17 to 50 years of age. Older users usually administer metaphedrone intravenously, while younger ones usually choose snorting and oral ingestion of the drug. In Serbia, metaphedrone is a legally controlled substance. The pharmacodynamic properties make metaphedrone similar to classical recreational drugs. The method of administration, mainly repeated administration in a single session, could be explained using the pharmacokinetic profile of the drug. The most reported symptoms of intoxication were those of a sympathomimetic nature, such as tachycardia, chest pain, hypertension, diaphoresis, and agitation. Most intoxications and fatal outcomes occurred to users who combined several psychoactive substances. The correlation between measured blood concentrations of the drug and outcomes of intoxication was not found. The mechanisms of metaphedrone’s toxicity are not fully understood. *Conclusions:* There is an increasing trend of abuse of metaphedrone among recreational drugs users. Future studies should focus on pharmacological and toxicological effects of metaphedrone on animals and humans.

## 1. Introduction

Metaphedrone (3-methylmethcathinone, 3-MMC) is a chemical substance widely described as an isomer of mephedrone (4-methylmethcathinone, 4-MMC), a compound that, in turn, belongs to a larger group of novel psychoactive substances called synthetic cathinones [1,2].

**Figure 1:**
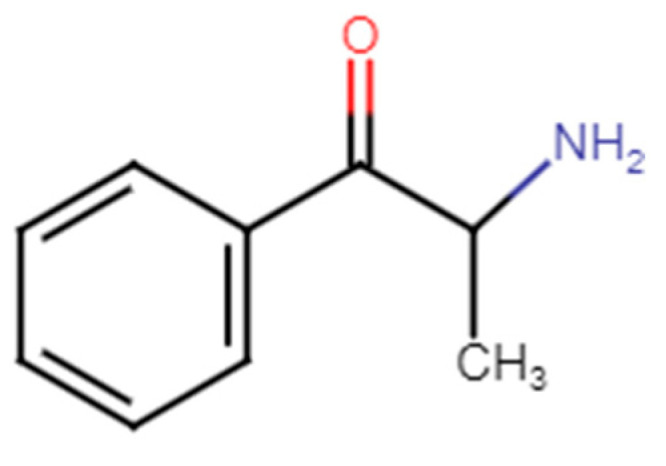
Cathinone [10].

**Figure 2:**
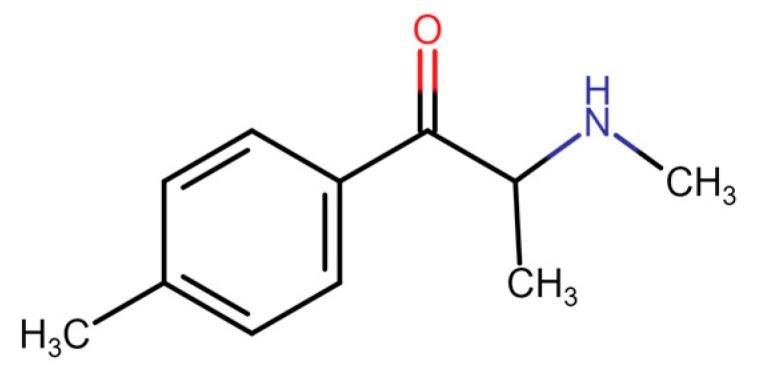
Mephedrone [10].

This group of structurally similar substances originates from cathinone, an amphetamine-like sympathomimetic amine. Cathinone (Figure 1) is the main psychostimulative substance naturally occurring in the leaves of the khat shrub (*Catha edulis*) [3,4]. This molecule was isolated from fresh leaves of khat in 1975 [5]. Prior to its isolation, several cathinone derivatives were already synthesized in the late 1920s, such as methcathinone (ephedrone) and 4-methylmethcathinone [6,7,8,9]. From then on, many other synthetic cathinones have been produced. The most popular and the best researched drug from this group of novel psychoactive substances is 4-MMC (Figure 2) [7].

Except for bupropion, a cathinone analog approved for clinical use as an antidepressant and smoking cessation therapeutic agent [11], it has not been shown that any other synthetic cathinones can be used for therapeutic purposes [1,12]. 

These compounds are marketed as “bath salts”, “plant food”, or “insect repellent”, and are labeled with “not for human consumption” or “research chemical” in order to be sold within legal boundaries [3,13,14]. They can be found in stores called “headshops” and on online shops. The mere fact that synthetic cathinones can be found on the Internet explains the widespread abuse of these drugs in the general population [6,15]. After one of these substances becomes legally controlled, which usually is the case, drug users start to buy it from dealers because it is no longer available to be obtained elsewhere [16].

After becoming a new trend in the recreational drug market, synthetic cathinones were initially considered as “legal highs”. Having had stimulating and emphatogenic actions, they were seen as alternatives for classical recreational drugs, such as amphetamine, 3,4-methylenedioxymethamphetamine (MDMA), and cocaine [17]. The lack of relevant literature regarding human toxicity and numerous reported cases of abuse, dependence, intoxications, and deaths raised significant concern when it comes to this group of novel psychoactive substances [6,18]. Therefore, over time, many of these substances became legally controlled. Being one of the most popular synthetic cathinones in terms of consumption, several countries banned 4-MMC by putting this substance under legal control, making it difficult to obtain for recreational purposes [1,3,19].

**Figure 3:**
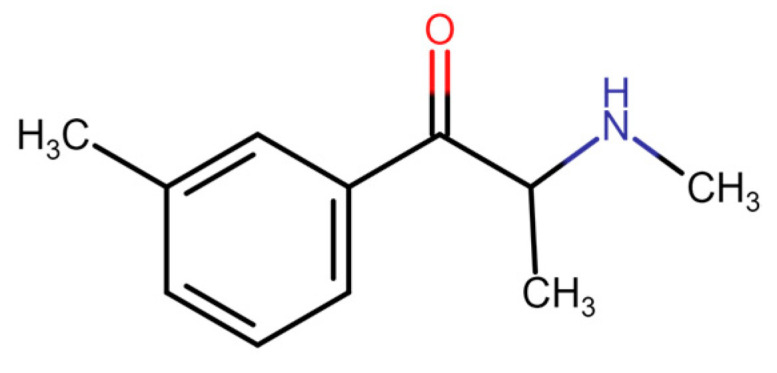
Metaphedrone [10].

As a consequence, a new generation of synthetic cathinones emerged on the market. Several new structurally related derivatives of 4-MMC gained their popularity over time and became available in the general population since it was possible to obtain them without legal consequences. They quickly became popular, and their abuse became an important public health issue in a short period of time [1,13]. Accordingly, this led to the detection of novel synthetic cathinones from samples of biological material or those obtained from drug markets. One of these substances was exactly 3-MMC (Figure 3) [2].

Metaphedrone is a structural isomer of mephedrone (4-MMC) and a psychostimulant drug [20,21,22]. This substance first appeared in Sweden in 2012 with the aim to replace 4-MMC after it was banned in the country [23]. In Poland, 3-MMC was introduced in 2013 to replace butylone and MDPV (3,4-methylenedioxypyrovalerone), both of which are also synthetic cathinones. In the year that followed, it became the most popular synthetic cathinone among drug users in Poland [24,25]. In Slovenia, a group of authors showed that the most frequently used novel psychoactive substances in 2014 were synthetic cathinones such as 3-MMC. This drug quickly replaced 4-MEC (4-methylethcathinone) and methylone, both of which replaced 4-MMC after its ban in the summer of 2011 [16]. The popularity of metaphedrone also grew rapidly in other European countries [1,23]. In the years that followed, 3-MMC did not become less popular. Moreover, the findings about 3-MMC gaining popularity over time are supported by the results that van Lonkhuyzen et al. came up with in their paper about 3-MMC poisonings [3]. Their retrospective study on the quantity of self-reported 3-MMC poisonings showed that the number of reports increased over time, from 2013 to June 2021 [3]. Presumably, the COVID-19 pandemic probably had a significant influence on the increase in the number of 3-MMC users during that period [3,26,27]. Considering the reduced accessibility (e.g., closing of borders limiting the usual supply) to usual recreational drugs which led to the popularization of online markets of novel psychoactive substances, it is believed that home use of these compounds was the predominant drug consumption pattern after the preventive and control measures were imposed [3,27,28]. Interestingly, the analysis of illicit drug seizures in Italy during the COVID-19 pandemic showed that 3-MMC was the most prevalent compound from the obtained samples of the seized drugs, thus indicating a correlation between the circumstances of the pandemic and the increased frequency of 3-MMC abuse [29].

The aim of this paper is to analyze and review the relevant research on the novel psychoactive substance, metaphedrone, as well as to present a pharmacological, clinical, and toxicological profile of the abovementioned compound.

## 2. Methodology

**Figure 4:**
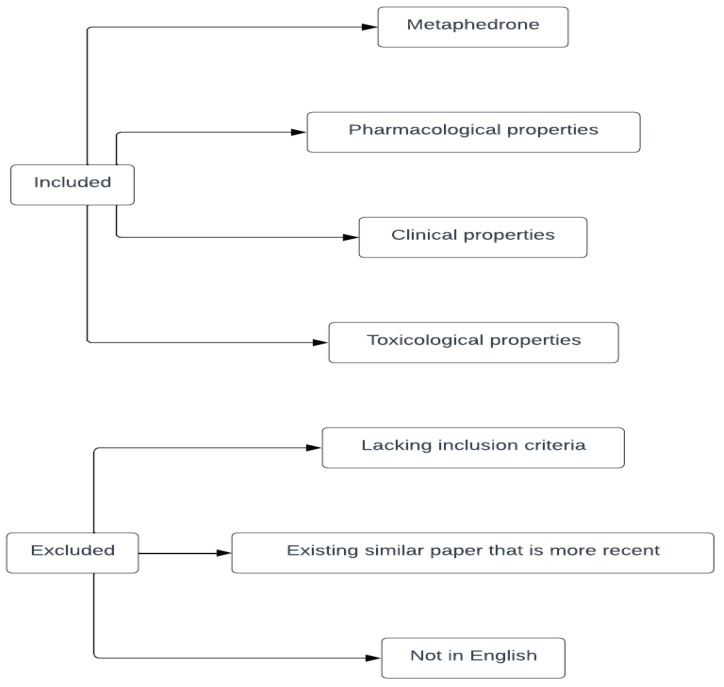
The qualities of the paper that were relevant for the decision on including or rejecting the studies. Note: an exception was made to the third exclusion criterion regarding references No. 44 and 50 due to the information that was relevant to the topic. Created online at: https://lucid.app/. URL (accessed on 27 February 2024).

The collection of relevant information about metaphedrone was performed by analyzing the relevant literature which was discovered with the aid of the search engines Google Scholar and PubMed. The keywords that were used to find the appropriate literature were *3-methylmethcathinone*, *3-MMC,* and *metaphedrone.* The search was not limited to a specific time period. A total number of 85 papers came up as a result. All of the topics that were mentioned previously (metaphedrone and its pharmacological, clinical, and toxicological profile) served as the inclusion criteria during the literature research. These areas of focus were afterwards thoroughly analyzed and presented. The material that was related to none of the abovementioned subjects was rejected and excluded from the analysis. The qualities that influenced the inclusion or exclusion of the obtained material can be seen in Figure 4.

Since this paper is a review article, it was not required for the Ethical Committee to provide an ethical approval.

## 3. Key Results and Discussion

### 3.1. Metaphedrone (3-Methylmethcathinone, 3-MMC)

According to the previously mentioned facts, this synthetic structural analog of mephedrone (4-MMC) became popular among users of novel psychoactive substances after legal prohibition of older generations of synthetic cathinones. Having become widely available, metaphedrone became a new, relatively easily accessible, psychoactive substance in the population of recreational drug users.

This novel psychoactive substance can be found online or bought from local drug dealers [1,9,16]. In Slovenia it is marketed as “*sladoled*” or ice cream, probably because this drug has a sweet scent [16], and what is more to physical properties of this drug is its characteristically licorice-like smell [20].

The most usual forms in which 3-MMC can be bought are white powder and crystal [30]. Metaphedrone is also formulated as a tablet or a capsule, according to the literature. However, a liquid form of the drug is also reported [1,3]. The packages containing metaphedrone are often labeled as “research chemical” and “not for human consumption” [31]. The most common methods of administration of 3-MMC are snorting (insufflation) and oral ingestion [1,13,16,23,24,30], while intravenous application was found to be not as common as the first two methods [1,23,24,30]. Inhalation (smoking) and rectal administration are rarely reported. Some individuals who abuse 3-MMC use a combination of methods of administration during single sessions [3,20,23].

All novel psychoactive substances are generally used because of their potent stimulatory, hallucinogenic, dissociative, euphoric, and/or relaxing properties [23]. The users of metaphedrone opt for this novel psychoactive substance for recreational purposes and/or for sexual stimulation [3,26]. The latter refers to chemsex/slamsex, a very serious public health concern, which is defined as an activity which involves the use of a drug or a combination of drugs to enhance sexual practices and sensations [3,26,32]. This activity became a recent phenomenon concerning the community of men who have sex with men (MSM) [21]. Sometimes, 3-MMC is purchased solely due to the shortage of traditional psychoactive drugs, such as MDMA [16]. The effects of 3-MMC are described by users of this substance as similar to those of MDMA and 4-MMC, but less intense [1,24]. Those effects are euphoria, excitement, high energy levels, stimulation (rushing), happiness, awareness of senses, unbridled libido, and an appreciation of music [1,17,21,24]. The social effects consist of improved social skills and feelings of empathy, but chronic abuse can lead to negative consequences in terms of close relationships with family, partners, and friends [1,24]. It was confirmed by a group of authors from China that metaphedrone has addictive potential in terms of its capability to cause significant psychological dependence after chronic exposure of drug users to 3-MMC [33]. The longer the abuse of this drug lasts, the higher the risk of dependence becomes [21].

The desired effects of 3-MMC appear between 15 min and 1 h, and they wear off after 4 to 6 h [21]. The short half-life of 3-MMC may be responsible for the short duration of its desired effects, so the repeated administration in one session is a common occurrence with consumers of this psychoactive substance [1,13]. Since information about the standard dosing per session to achieve the desired effects does not exist, drug users have to rely on subjective information from Internet chat forums for psychoactive substances. This information is surely far from scientifically proven facts, but these drug forums serve as important sources of information for researchers who are investigating the toxicity of 3-MMC and aiming to determine the threshold toxic and lethal doses [15]. Users of 3-MMC reported various drug administration doses, most frequently ranging from 50 to 500 mg [24]. Nevertheless, larger amounts up to 1000 mg were reported on some occasions. A group of researchers from Sweden reported the dose range from 0.5 to 2 g [23]. Taking into consideration sessions with repeated administrations, the doses sometimes reached up to 6 g in a single session [1,3,24]. Additional toxicological analyses are required in order for the precise dosing information to be provided to the users so that the potential risks associated with the overdose (e.g., intoxications ranging from mild to fatal, chronic organ damage, dependence, and tolerance) could be reduced to a minimum or even eliminated.

Several studies in Europe showed that the most frequent users of metaphedrone were males from 17 to 50 years of age [1,3,23,24,31,34]. However, a group of authors from Slovenia found no gender differences in the drug consumption patterns, with most of the respondents still attending school. This drug is commonly used in this age group probably due to its low price [16]. Moreover, the data found in several reports suggest that intravenous users are generally older than those who expose themselves to 3-MMC by snorting or oral ingestion [3].

There are scientific reports that pointed to a reduction in exposure to 3-MMC after the legal prohibition of the drug. Ledberg [35] studied the curiosity in novel psychoactive substances using a Swedish Internet discussion forum to track the interest by counting the number of posts per day. When it came to 3-MMC, this number dropped dramatically after the prohibition of the drug in relation to the number of daily posts when the drug was not legally controlled [35]. The results from the Swedish STRIDA project showed that the exposure to 3-MMC decreased after the substance became legally controlled in this country [23]. The legal status of 3-MMC is variable in different countries. It is a controlled psychoactive substance in many European countries, the United States and China, but in several countries, it is still legally available and can be found online or in headshops [1,12].

In Serbia, metaphedrone is still a novel psychoactive substance that lacks relevant information that would enable a better understanding of its regional prevalence of abuse, availability, means of obtaining the drug, and the demographic characteristics of users of this substance. The legal status of 3-MMC in Serbia (“RS Official Gazette”, No. 4/2020) is that of a psychoactive controlled substance that is still not classified in accordance with the Customs Tariff Law (CAS number: 1246816-62-5). According to the decision on specification of goods for which import, export or transit is prescribed to procure specific documents (“RS Official Gazette”, No. 59/2022, 107/2022, 3/2023 and 27/2023), 3-MMC is classified as the “psychotropic substance for which import, export, or transit is necessary to procure a permit”. The abovementioned statements imply that metaphedrone is a substance that is legally controlled in Serbia.

### 3.2. Pharmacological Profile

Luethi et al. performed a study to characterize in vitro pharmacology of novel analogs of mephedrone (4-MMC) and related newly emerged designer stimulants. Among other mephedrone analogs, they obtained results on the pharmacological profile of 3-MMC [22].

**Figure 5:**
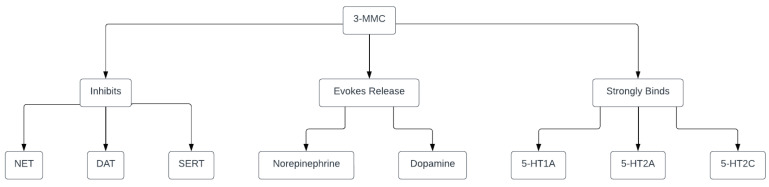
Mechanism of action of metaphedrone. Legend: 3-MMC-3-methylmethcathinone; NET-norepinephrine transporter; DAT-dopamine transporter; SERT-serotonine transporter; 5-HT1A, 5-HT2A, 5-HT2C-serotonine (5-hydroxytriptamine) receptor subtypes. Created online at: https://lucid.app/. URL (accessed on 27 February 2024).

*Pharmacodynamics.* According to their findings, 3-MMC potently inhibits the norepinephrine transporter (NET) which is probably responsible for sympathomimetic stimulation and cardiovascular adverse effects, being similar, in terms of its effects, to amphetamine and MDMA. 3-MMC more potently inhibits the dopamine transporter (DAT) than the serotonin transporter (SERT). These results suggest that 3-MMC has stronger amphetamine-like psychostimulant properties than mephedrone and its structural analogs. Like most amphetamines and other synthetic cathinones, 3-MMC is also a substrate-type monoamine releaser and evokes the release of norepinephrine and dopamine in the synaptic cleft. Also, this drug strongly binds to the serotonin 5-HT_1A_, 5-HT_2A_, and 5-HT_2C_ receptors, similar to mephedrone and MDMA, but also with a significant difference since it does not activate 5-HT2A receptors, thereby the substance seems to be more of a stimulant than a hallucinogen (although in certain regions it has been classified as a hallucinogen for regulatory purposes) [22]. The mechanism of action of 3-MMC is visually presented in Figure 5.

*Pharmacokinetics.* Little data are available on the pharmacokinetics of 3-MMC [1]. In the current literature, there are no existing human studies on this topic. In their research, Shimshoni et al. investigated and characterized a pharmacokinetic profile of metaphedrone by administering it to domestic pigs intravenously and orally [13]. The greatest portion of the drug (more than 80%) was absorbed in the first 12 min after oral ingestion and it was calculated that the mean oral bioavailability was 7%, which suggests that this drug undergoes an extensive first-pass effect [36]. The low oral bioavailability might explain why insufflation is a more common way of ingesting this substance. Being reported as a route of administration of 3-MMC, it needs to be mentioned that the bioavailability of this drug is expected to be higher when administered rectally because it then enters systemic circulation without first passing through the liver [20,37]. 3-MMC is well distributed into the extravascular tissues with a volume of distribution of 240 L. This substantial value might be explained by low protein binding and the extensive active transport into tissues. However, it was determined by the analysis of the brain samples of the pigs that there was a lack of accumulation and rapid elimination of the drug from the central nervous system. Similar to mephedrone (4-MMC), the metabolism of metaphedrone (3-MMC) in humans still lacks significant information. From what is known so far, 3-methylephedrine and 3-methylnorephedrine are the metabolites of this drug [1,38]. In addition to the available data on metabolites of 3-MMC, Aknouche et al. identified desmethyl-3-MMC and hydroxyl-3-MMC as another two metabolites of 3-MMC. Their analysis of biological samples of a deceased individual in their research also showed traces of carboxy-3-MMC in the urine of the decedent [20]. The common metabolic pathways for 3-MMC might be similar to 4-MMC, and those are ß-keto-reduction and N-demethylation [39]. Metaphedrone’s extensive total clearance of 199 L/h results in a very short half-life of 0.83 h. Consequently, it was shown that the drug disappears from plasma after 4 h. This finding suggests that there might be other elimination sites/mechanisms besides the liver and kidneys that contribute to the elimination of 3-MMC, since the sum of liver and kidney blood flow was 88 L/h in pigs that weighed 30–40 kg [13].

### 3.3. Clinical Profile

Despite the aforementioned desired effects, the use of 3-MMC often also comes with the acute adverse effects which can be divided into four groups of signs and symptoms: cardiovascular, neurologic, psychiatric, and other signs and symptoms of 3-MMC intoxication [3]. The most frequently reported symptoms were those of a sympathomimetic nature such as tachycardia, chest pain, hypertension, agitation, and perspiration [1,3,24]. Commonly reported signs and symptoms of cardiovascular origin were tachycardia, hypertension, chest pain, palpitations, ECG abnormalities, and cardiac arrest. Neurological signs and symptoms include verbosity, stuttering, slurred speech, fatigue, drowsiness, reduced level of consciousness, uncoordinated movements, staggering, tingling in the arms and legs, bruxism, dilated pupils, headache, dizziness, insomnia, and convulsions. Psychiatric phenomena include difficulties with concentration, confusion, disorientation, fear, anxiety, depression, agitation, aggression, paranoid delusions, and hallucinations. Other signs and symptoms of 3-MMC intoxication are gastrointestinal distress, diaphoresis, dyspnea, tachypnea, dry mouth/throat/nose, hyponatremia, hyperthermia, and rhabdomyolysis [1,3,16,21,23].

If 3-MMC is combined with other substances, more serious adverse effects and florid clinical pictures can be expected [1,16,23,24]. The listed adverse effects are similar to those of 4-MMC and MDMA. However, the effects of 3-MMC appear less intense and shorter in duration [3,16]. For these patients, it is important to take into consideration and check for potential frequent comorbidities, such as hepatitis C or HIV [20,23,40].

To carry out blood analysis for 3-MMC, when aiming for the detection of the drug(s) responsible for the intoxication, advanced analytical methods are required. A liquid chromatography-tandem mass spectrometry (LC-MS/MS) is standardly performed because this method is considered suitable for the toxicological analysis of biological specimens [1,3,24].

### 3.4. Toxicological Profile

In general, the clinical data on 3-MMC poisoning are scarce [1,21,41]. The available data on toxicity of 3-MMC contain reports about fatal and non-fatal intoxications that happened in relation to the abuse of 3-MMC [1,12]. One of the main questions from a toxicological point of view is the one concerning the toxic and lethal concentrations of 3-MMC. The consensus of a threshold of toxic and fatal concentration in humans has not been reached yet. It was found that there appears to be no correlation between measured concentration and the outcome of intoxication (fatal or not). The reasons for this might be the chemical instability of the drug, the lack of literature and the current absence of controlled human studies. Due to its chemical instability, an underestimation of blood concentration is possible [21]. The detected blood concentration might also be incongruent to the number and intensity of symptoms due to the tolerance phenomenon [24].

Various reported measured concentrations of metaphedrone in the blood of intoxicated individuals support the abovementioned claims. One accidental intoxication occurred in France in 2017, when a young man died a couple of hours after sniffing an unknown substance. The analysis of postmortem peripheral blood revealed 3-MMC at a concentration of 249 ng/mL. The cause of death was monointoxication with 3-methylmetcathinone [42]. Ameline et al. reported two fatal cases that tested positive for metaphedrone at 613 and 462 ng/mL [21,30]. Adamowicz et al. performed an analysis of 95 cases where 3-MMC blood concentrations were ranging from less than 1 ng/mL up to 1600 ng/mL. From their sample, the highest concentration of 3-MMC (1600 ng/mL) was measured from blood collected during the autopsy of 20-year-old man who co-ingested 5-(2-aminopropyl)benzofuran (5-APB) and ethyl alcohol, whose concentrations were, respectively, 5.6 µg/mL and 1.4 g/L [17,24]. In 2022, Aknouche et al. reported a case of a fatal outcome of 3-MMC poisoning in the context of chemsex, where a blood concentration of 3-MMC was measured at 1437 ng/mL [20]. The reports on two fatal intoxications in Italy in 2015 and France in 2016 showed the following concentrations of metaphedrone, respectively: 249 ng/mL and 4400 ng/mL [43,44].

3-methylmethcathinone is a drug sometimes used for suicidal purposes according to some reports. An observation was made by a group of British authors that there seems to be a relationship between the use of synthetic cathinones and suicidal behavior. In a fatal case of a suicide poisoning of a 19-year-old female with 3-MMC, a toxicological analysis of blood revealed 3-methylmetcatinone at the concentration of 800 ng/mL [41,45].

Due to the fact that the desired effects of 3-MMC do not last long, users usually increase the concentration of 3-MMC by repeating the administration in a single session, which may escalate the risk of overdose [24]. Most of the reported deaths and intoxications happened because users mixed 3-MMC with one or several other psychoactive substances [1,17,23,24,30,42,43,44,45]. For instance, Jamey et al. published a case of lethal poisoning of a 69-year-old male with 3-MMC, gamma-hydroxybutyric acid (GHB), and pseudoephedrine, revealed by a toxicological analysis at the following concentrations, respectively: 330 ng/mL, 576 ng/mL, and 33 ng/mL [30]. Another example is the case of intoxication of a 31-year-old man from France, involving 3-MMC and GHB during a chemsex session. The measured blood concentrations were 117 ng/mL and 131 mg/L, respectively [26]. A study from Sweden found that polydrug users combined 3-MMC with another novel psychoactive substance or more of them, or they used metaphedrone with traditional psychoactive substances, such as amphetamine, buprenorphine, cannabis, and ethanol [23]. The combined abuse of narcotics is one of the main reasons why toxic and lethal concentrations of 3-MMC cannot be determined easily.

The mechanisms of toxicity of 3-MMC are not clarified to the full extent. Only several hypotheses are currently proposed. Being structurally similar and having similar mechanisms of action to amphetamine and MDMA, it is assumed that 3-MMC might share some mechanisms of toxicity with these substances [1,46,47,48]. Like amphetamine, 3-MMC shows the capability to induce hyperthermia, which was described by Bäckberg et al. [23]. Having inhibitory properties towards serotonin transporter function, metaphedrone can lead to the accumulation of excess serotonin in the synaptic cleft. As a consequence of this effect, the risk of developing hyperthermia significantly increases [1,41,49,50]. Bäckberg et al. reported a lethal case of 3-MMC poisoning where a patient had severe hyperthermia (40.9 °C) which lasted up to 20 h. The hyperthermia was refractory to sedation, external cooling, and the administration of cold fluids. The renal function of the patient gradually deteriorated as a result of the development of metabolic acidosis and rhabdomyolysis. After six days, the patient was pronounced dead [23]. In relation to the abovementioned, having been described as an adverse reaction to the abuse of synthetic cathinones, it can be expected that serotonin syndrome could potentially develop in individuals who abuse 3-MMC [50,51].

Da Silva et al. [52] conducted a study aimed at assessing the hepatotoxicity of 3-MMC using primary rat hepatocytes. According to the fact that the liver has an important role in cathinones’ metabolism [46], particularly after administered *per os*, it is of current scientific opinion that metaphedrone undergoes bioactivation that produces metabolites with higher toxicity, which are believed to be capable of inducing harm to the functioning of the liver and thus to the overall homeostasis of the intoxicated individual [52]. The results of their investigation showed that the liver is indeed sensitive to 3-MMC-induced toxicity and the extent of damage to the cells seems to be directly proportional to the concentration of the drug to which they were exposed. The CYP450 enzymes, especially CYP2D6 and CYP2E1, were shown to play an important role in the biotransformation of this substance [52]. The polymorphisms of genes encoding these enzymes, primarily CYP2D6, play a significant role in individual responses to the ingested drug. The severity of intoxication may vary depending on the allele that an individual carries. Therefore, it can be expected that “extensive and ultrarapid metabolizers” will suffer a more severe 3-MMC poisoning than “poor and intermediate metabolizers” [53]. Similar to amphetamine, metaphedrone also increased intracellular oxidative stress through the production of reactive oxygen and nitrogen species after exposing the hepatocytes of experimental animals to the drug. Consequently, intracellular glutathione depletion was observed, implying that liver cells are capable of defending themselves against the toxic effects of 3-MMC. However, if the toxicity of 3-MMC prevails over the cells’ defensive capabilities, intrinsic, extrinsic, and common apoptotic pathway activation occurs as a reaction, which was also proven in this study [52]. This research represents an important checkpoint in the research concerning 3-MMC. Therefore, further investigation of this drug’s toxicity is required in order to fully understand the risks associated with the abuse of this novel psychoactive substance.

## 4. Conclusions

According to information from the relevant literature, we can conclude that there is a growing trend of abuse of metaphedrone among recreational drug users. Hence, further investigations into this novel psychoactive substance are required. Future studies should primarily focus on pharmacological and toxicological effects of metaphedrone on animals and humans. In order to obtain more information on the prevalence and characteristics of the use of 3-MMC in our area, specifically designed questionnaires should be given at places where individuals who abuse drugs are expected to be present (e.g., night clubs or addiction departments at psychiatry clinics). The results of the proposed studies would potentially be of great significance in determining whether the abuse of metaphedrone should be considered a potential public health problem or not.

## Data Availability

Data are contained within the article.
